# Efficacy of regular professional brushing by a dental nurse for 3 months in nursing home residents—A randomized, controlled clinical trial

**DOI:** 10.1111/idh.12389

**Published:** 2019-02-22

**Authors:** Anna Greta Barbe, Hannah E. Kottmann, Sonja H. M. Derman, Michael J. Noack

**Affiliations:** ^1^ Department of Operative Dentistry and Periodontology, Centre of Dental Medicine University of Cologne Köln Germany

**Keywords:** dentistry nursing, gerodontology, nursing home, oral hygiene

## Abstract

**Objectives:**

The oral health of nursing home residents is poor for various reasons. Many require help for oral hygiene. Regular professional brushing by a dental nurse should improve oral hygiene. This study aimed to determine the efficacy of regular tooth brushing by a dental nurse on the oral health of nursing home residents.

**Methods:**

This controlled trial randomized participants (n = 50; mean age 83 ± 8 years) to brushing by a dental nurse every 2 weeks for 3 months (n = 25; test group) or oral hygiene procedures performed/controlled by nursing home staff (n = 25; control group). Personal, general and oral health, as well as various oral hygiene parameters—plaque index (PI), gingivitis index (GI), papilla bleeding index (PBI), oral hygiene index (OHI) and Volpe‐Manhold Index (VMI)—were evaluated at baseline, after initial professional dental cleaning and before last brushing.

**Results:**

At baseline, oral health was impaired according to investigated indices in both groups. After professional brushing for 3 months, there were improvements in PI, GI and PBI, with significant increases compared with the control group in OHI and VMI (*P* = 0.017 and *P* < 0.001, respectively). Among the control group, the number of teeth decreased while the root caries index increased (*P* = 0.002 between groups).

**Conclusions:**

Regular professional brushing every 2 weeks by a dental nurse can be recommended for nursing homes residents to improve oral health parameters and to help reduce root caries incidence as a basis to preserve the number of teeth. Such oral hygiene procedures will maintain and improve the oral health of nursing home residents.

## 1 INTRODUCTION

The number of people that depend on care in nursing homes will increase dramatically over the next few decades. In 2009, over two million inhabitants in Germany alone needed such care.^1^ The oral health of nursing home residents has been well documented and is poorer than that of older community‐dwelling people. In particular, periodontitis, root caries, dry mouth and existence of prostheses have shown higher prevalence.^2^, ^3^ Furthermore, oral care in nursing homes is inadequate, aggravated by the lack of awareness of good dental hygiene practices among nursing staff.^4^, ^5^, ^6^, ^7^, ^8^, ^9^, ^10^ Various systemic illnesses, multi‐morbidities and frequent polypharmacy in nursing home residents can exacerbate the situation. Additionally, nursing home residents often suffer from dementia and immobility, which may hinder them and the caring staff from performing good oral hygiene procedures.^11^, ^12^ It has been suggested that oral hygiene performed in nursing homes is insufficient for the maintenance of good oral health.^4^ The fact that somebody requires external help for oral hygiene manoeuvres may itself represent an increased risk of impaired oral health. Therefore, new and realistic strategies must be developed to improve the oral health of this special population.

One solution could be to provide regular professional brushing performed by a dental nurse, alongside other methods, to improve oral care, achieve constant long‐term oral health benefits and potentially improve quality of life. The purpose of our randomized, controlled clinical study of nursing home residents was to investigate the impact of professional brushing performed every 2 weeks by a dental nurse on the number of teeth (main outcome parameter), incidence of root caries, and further short‐term oral health parameters, compared with residents whose oral hygiene was performed or supervised by staff according to standards of care corresponding to German law concerning the care for the elderly. We hypothesized that regular professional brushing would be efficient in maintaining or improving individual oral health status.

## 2 MATERIALS AND METHODS

### 2.1 Ethics

The University of Cologne local ethics review board (16‐204) granted approval for the study. The study was registered under DRKS00010767 at the German Clinical Trials register (https://www.germanctr.de) before the first patient was enrolled. All procedures performed were in accordance with the ethical standards of the institutional research committee and with the 1964 Helsinki declaration and its later amendments or comparable ethical standards. Informed consent was obtained from all individual participants included in the study or their legal guardians.

### 2.2 Subjects

Fifty‐one nursing home residents (St. Elisabeth nursing home, Bornheim, Germany) or their legal advisors were asked between August 2016 and October 2017 to participate in the study; of these, 50 were willing and were included after written informed consent was obtained. The nursing home has a cooperating dentist from whom all inhabitants receive dental care on a regular basis according to standards of outpatient dental care in Germany. All participants had regular medical and dental health institutional insurance.

Patients were excluded if they had <4 remaining teeth, a life‐threatening condition at risk of imminent demise of the resident and conceivable loss of the remaining teeth (eg because of diagnosed inflammation with apical osteolysis or loosening with mobility of over 2 mm in buccolingual direction and intrusion of teeth according to Grade 3 of the Grace & Smales Mobility Index).

### 2.3 Study design and procedures

Personal health parameters were obtained and documented from the medical files of the nursing home, including care dependency level (according to German law concerning the care for the elderly, care dependency is classified into grades 1‐5, from 1 = little impairment of independency to 5 = highest impairment of independency with special needs for nursing care); number of months living in the nursing home, mobility and oral hygiene practices (self‐brushing with a manual toothbrush or using interdental devices (usually interdental brushes) without supervision by staff, brushing by staff with a manual toothbrush or supervision of resident's brushing with a manual toothbrush by staff); and general health parameters (including cognitive status, prescribed medications, systemic diseases). Study participants as unit of randomization were randomized to either the treatment or the control group. A block randomization was carried out and the block size varied with a maximal number of four. Afterwards, all participants received a baseline oral examination by the cooperating dentist, followed by professional dental cleaning to reach a standardized level of oral hygiene. An additional oral examination was then performed to document cleaning success and acceptance among study participants. Subsequently, nursing home staff received in‐house training regarding oral hygiene knowledge and practice guidelines for nursing home residents.

**Figure 1:**
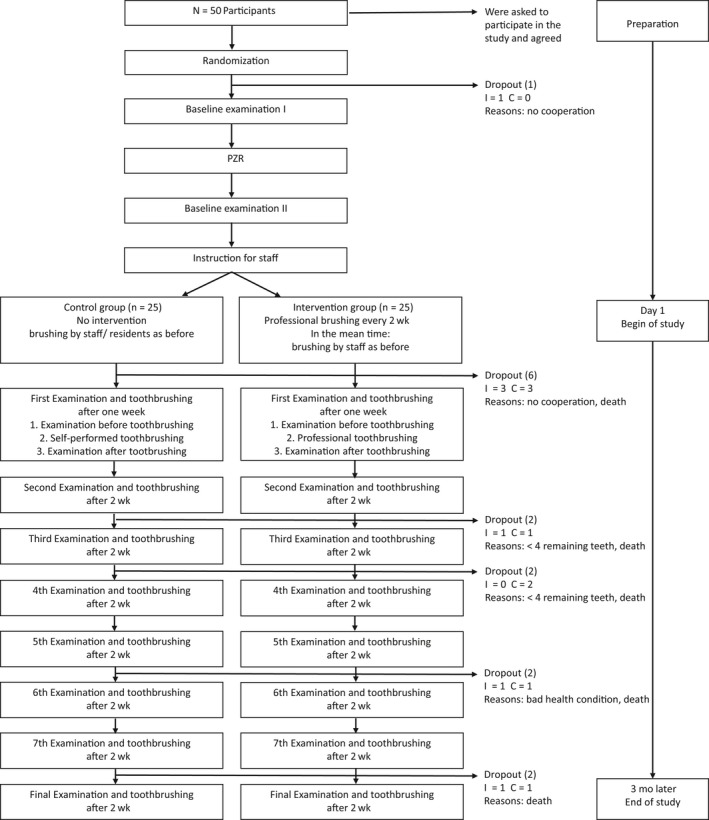
Study flow chart

During the intervention phase, the control group received oral hygiene procedures performed or controlled by the nursing home staff (treatment as usual). In the treatment group, a dental nurse performed one brushing session every 2 weeks. Also every 2 weeks, every study participant received an oral examination; the final examination was performed after 3 months, directly before the final brushing session. A study flow chart, including dropouts, is shown in Figure 1. The dental nurse was instructed and calibrated according to the educational guidelines for dental students of the Medical Dental University of Cologne.

#### 2.3.1 In‐house training

Before the start of the intervention phase and after performing professional dental cleaning for all study participants, all nursing home staff received an in‐house training session regarding oral hygiene recommendations for daily practice in nursing homes. This was done to provide standardized knowledge regarding providing oral hygiene for residents, supervising resident's brushing or controlling results of self‐brushing residents. Residents did not receive any additional training, since clean results were supervised by the educated staff. First, the cooperating dentist of the nursing home held one educational session regarding the general necessity of regular oral hygiene provision to nursing home residents, and the actual oral hygiene and oral health situation in this nursing home. Second, a standardized lecture regarding oral health and oral hygiene among older people, distributed by the German Society of Gerodontology,^13^ was held. Afterwards, instructional films were shown addressing correct oral hygiene procedures, prosthetic reincorporation and replacement, and their correct cleaning procedures (https://www.bzaek.de/fuer-medien/video-audio.html, last assessed 15 November 2018). Finally, jaw models and toothbrushes were used to perform practical exercises.

#### 2.3.2 Professional dental cleaning (initial pre‐intervention procedure)

At the cleaning appointment, all participants were visited in their rooms by the dental nurse. First, the nurse prepared the necessary equipment (scaler, brushes, ultrasonic cleaning device, interdental brushes) in a central bathroom. Afterwards, participants walked or were transported to the central bathroom where they were seated and the oral examination was completed. Professional dental cleaning was performed with brushes, ultrasonic cleaning, scalers and interdental brushes until a macroscopic clean situation was reached controlled and documented by the cooperating dentist. If participants refused to continue the procedure, the nurse would stop immediately (nobody did).

#### 2.3.3 Control group

Among study participants in the control group, besides in‐house training of nursing home staff and pretreatment in form of the professional dental cleaning session, no other parameters were changed regarding their oral hygiene practices from before study participation. Oral hygiene practice in the control group was self‐brushing with a manual toothbrush or using interdental devices (usually interdental brushes) without supervision by staff, brushing by staff with a manual toothbrush or supervision of resident's brushing with a manual toothbrush by staff.

#### 2.3.4 Brushing group

One mouth mirror, the toothbrush (Oral B Pro‐Expert CrossAction, Schwalbach, Germany) and the toothpaste (Elmex Caries Prevention Professional; GABA, Hamburg, Germany) were prepared prior to the brushing session. Because of its mild taste, we assumed it would be well accepted by the participants, and 1450 ppm provided sufficient fluoride supply. Additionally, interdental brushes were available in different sizes. First, removable dentures were taken out of the mouth and cleaned. In advance, participants rinsed their mouth with water to remove food debris. The dental nurse placed residents on a chair and performed brushing from behind, supporting the resident's head with her arm. Occlusal surfaces were brushed first. For cleaning of the other surfaces, the modified Bass technique was applied.^14^ After again rinsing the mouth with water, interdental brushes adapted to interdental spaces were used. The length of denture brushing did not exceed 3 minutes, and dental brushing did not exceed 3 minutes according to timeframes specified from the nursing director of this nursing home for self‐brushing, while the use of interdental brushes did not exceed an additional 2 minutes.

### 2.4 Outcome parameters assessed

#### 2.4.1 Oral examination

The total number of teeth and prostheses prevalence were documented before professional dental cleaning, before the first dental brushing session, and before and after the last tooth brushing session. The periodontal status according to the community periodontal index of treatment needs (CPITN)^15^ should also be documented at these timepoints; because of missing participants’ compliance, CPITN was only documented before professional dental cleaning.

#### 2.4.2 Dementia status

The exact diagnosis of dementia was inconsistently documented in the nursing home. For some residents, information on dementia type and grade was documented, but often this information was missing. In this nursing home, on the day of moving in, residents are assigned to rooms according to the information “dementia yes/no,” based on the available diagnoses made by a neurologist. When planning this study, we decided to use this available binary assignment for all participating residents.

#### 2.4.3 Indices

Plaque index (PI),^16^ gingivitis index (GI),^17^ Quigley‐Hein index (QHI)^18^ and the papilla bleeding index (PBI)^19^ were obtained as described in detail elsewhere. The root caries index (RCI) was graded on a scale from RC1 (hard surface) to RC5 (soft surface),^20^ and the dental nurse was calibrated according to earlier approaches.^21^ Oral hygiene was documented by the oral hygiene index (OHI)^22^ and the amount of calculus by the Volpe‐Manhold Index (VMI).^23^ Indices were obtained before professional dental cleaning, before the first brushing and before the last brushing session.

#### 2.4.4 Xerostomic visual analogue scale (VAS)

All participants were asked “How dry is your mouth?” independent from their cognitive status, and answers were recorded as continuous variables from 0 cm = “not dry at all” to 10 cm = “no saliva at all.”^24^ No objective salivation rates were investigated.

#### 2.4.5 Food debris (vestibulum, upper prostheses, lower prostheses)

Food debris in the vestibulum was documented after rinsing once with water via a six‐grade scale. For participants without prostheses, the scale contained from 1 = no food debris, 2 = smallest singular pieces of food debris, 3 = vestibulum covered up to one‐third with food debris, 4 = vestibulum covered up to two thirds with food debris, 5 = vestibulum covered completely with food debris and 6 = vestibulum covered up to occlusal surface with food debris. If participants had prostheses, food debris was also documented via a six‐grade scale from 1 = no food debris, 2 = smallest singular pieces of food debris not reaching prosthetic teeth, 3 = prosthetic teeth covered up to 25% with food debris, 4 = prosthetic teeth covered up to 50% with food debris, 5 = prosthetic teeth covered up to 75% with food debris and 6 = prostheses completely covered with food debris up to occlusal surfaces. The maximum reached index number was documented and taken for statistical analysis.

### 2.5 Data analysis

Absolute and relative frequencies are given for qualitative variables, and mean ± standard deviation (SD) are given for quantitative variables. Group differences were tested using unpaired *t* test or Fisher's exact test, respectively. According to the analysis pre/post‐mean and SD, mean difference with 95% confidence intervals (CI) or *P*‐values are presented. Regarding index differences between baseline examination, examination before first brushing and examination before last brushing, Wilcoxon signed rank test and Friedmann's test with alpha adjustment were performed. All reported *P*‐values are two‐sided and considered statistically significant if lower than 5%; at Friedmann's test after alpha adjustment, *P*‐values were considered statistically significant if lower than 1.7%. All calculations were done with SPSS Statistics 24 (IBM Corp., Armonk, NY, USA). Data were entered twice and reconciled in case of inconsistencies.

## 3 RESULTS

### 3.1 Clinical characteristics

Fifty nursing home residents or their legal advisors provided written informed consent and participated in the study (Table 1). Of the participants, 68% were female, the mean age was 83 (SD: 8) years, and the mean time spent living in the nursing home was 8 (SD: 8) months, with a care dependency level of 3 (SD: 1). All residents had medical and dental health insurance. Dementia was diagnosed by their neurologist in 70% of participants, 84% were mobile, and they used 8 (SD: 5) prescribed daily medications, while 5 (SD: 2) comorbidities were documented from the medical files. Overall, 81% of study participants reported that they would brush their teeth by themselves, 73% with and 100% without diagnosed dementia. However, participants with dementia received help or supervision by the nursing home staff. Regarding the dental clinical examination, participants had 17 (SD: 9) remaining teeth, the RCI was 1.5 (SD: 1.6), and 78% suffered from periodontitis. The mean xerostomic VAS value was 1 (SD: 2). 14% of participants had total removable prostheses of the upper or lower jaw, 48% had partial removable dentures, and 84% had fixed prosthodontics (Table 1). In demented residents, the mean PI was 2.7 (SD: 0.4), without any difference compared to non‐demented residents 2.4 (SD: 1). There were no differences in clinical characteristics between intervention and control group.

**Table 1:** Clinical characteristics

	Study participants, n (%)	Brushing group, n (%)	Controls, n (%)	*P*‐value
Gender				
Female	34 (68)	19 (76)	15 (60)	0.234
Demented	35 (70)	16 (64)	19 (76)	0.365
Mobile	42 (84)	22 (88)	20 (80)	0.451
Periodontitis	39 (78)	20 (80)	19 (76)	0.739
Total prosthesis upper or lower jaw	7 (14)	2 (8)	5 (20)	0.295
Partial removable dentures	24 (48)	13 (52)	11 (44)	0.146
Fixed prosthodontics	42 (84)	22 (88)	20 (80)	0.451
	Mean (SD)	Mean (SD)	Mean (SD)	
Age, y	83 (8)	84 (10)	83 (7)	0.802
Number of months in nursing home	8 (8)	9 (8)	8 (8)	0.895
Nursing grade (1‐5)	3 (1)	3 (1)	3 (1)	0.750
Comorbidities	5 (2)	5 (2)	5 (2)	0.897
Total number of APIs	8 (5)	8 (5)	9 (5)	0.242
Xerostomia, VAS	1 (2)	1 (2)	0 (1)	0.094
DMFT	14 (8)	14 (7)	13 (8)	0.401

APIs, active pharmaceutical ingredients; DMFT, Decayed Missing Filled Teeth Index; SD, standard deviation; VAS, visual analogue scale.

#### 3.1.1 Differences between residents with and without dementia

Residents with and without dementia showed differences in baseline xerostomic VAS (mean score on 10‐cm xerostomic VAS 1.2 [SD: 1.9] in persons with dementia vs 0 in persons without; *P* = 0.001, no clinical impact), and persons with dementia had more food debris in the vestibulum (mean vestibulum index score 2.7 [SD: 2.1] in residents with dementia vs 1.1 [SD: 0.6] in residents without dementia; *P* < 0.001), and on the upper prosthesis (mean upper prostheses index 1.9 [SD: 2.3] in residents with dementia vs 0.7 [SD: 1.3] residents without dementia; *P* = 0.037; Supplementary file 1).

### 3.2 Oral health indices

**Figure 2:**
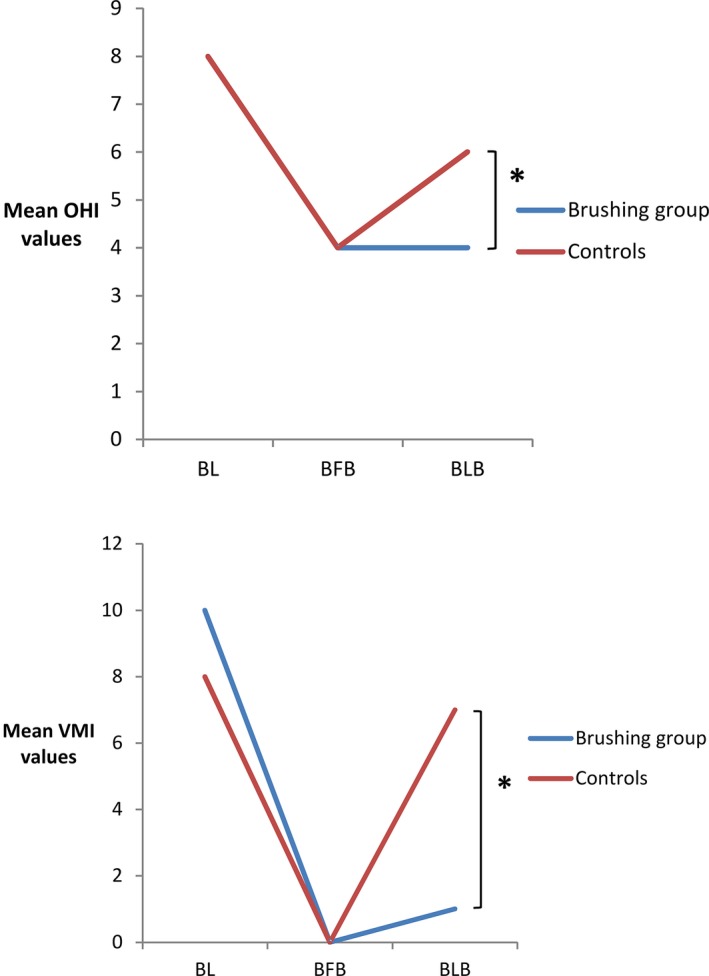
Mean Oral health index (OHI) and Volpe‐Manhold Index (VMI) values at baseline, before first brushing and before the last brushing session. *P < 0.017 after alpha adjustment; BFB, before first brushing; BL, Baseline; BLB, before last brushing

Table 2 illustrates the changes observed in oral health over 3 months. At baseline, mean indices were high in all patients. Two weeks after professional dental cleaning in all participants, but before the first interventional brushing, the indices were similar or had been reduced. After 3 months of professional brushing, there were significant reductions compared with baseline in PI (*P* = 0.027), GI (*P* = 0.008), OHI (*P* < 0.001) and VMI (*P* < 0.001). In the control group, significant reductions compared with baseline were recorded for GI (*P* = 0.013), OHI (*P* < 0.001) and VMI (*P* < 0.001). Significant between‐group differences were noted for OHI before last brushing (*P* = 0.017) and VMI before last brushing (*P* < 0.001), in favour of the intervention group (Figure 2).

**Table 2:** Investigated oral health indices at baseline, before first brushing and before the last brushing session

	Brushing group, mean (SD)	Controls, mean (SD)	*P*‐value
Plaque index			
Baseline	2.7 (0.5)	2.6 (0.6)	0.687
Before first brushing	2.7 (0.4)	2.5 (0.7)	0.225
Before last brushing	2.4 (0.6)	2.5 (0.8)	0.449
*P*‐value**	**0.027**	0.334	
Gingivitis index			
Baseline	2.3 (1.1)	2.2 (2.1)	0.607
Before first brushing	1.8 (1.1)	1.4 (1.1)	0.266
Before last brushing	1.4 (1.2)	1.7 (1.3)	0.597
*P*‐value**	**0.008** *	**0.013** *	
Quigley‐Hein index			
Baseline	4.2 (1.3)	3.9 (1.3)	0.524
Before first brushing	4.1 (1)	3.9 (1.5)	0.762
Before last brushing	3.9 (1.2)	4.1 (1.5)	0.609
*P*‐value**	0.150	0.819	
Papilla bleeding index			
Baseline	2.6 (1.5)	2.2 (1.5)	0.382
Before first brushing	2.3 (1.1)	1.9 (2.2)	0.488
Before last brushing	1.7 (1.4)	2.1 (1.8)	0.336
*P*‐value**	0.076	0.568	
Oral hygiene index			
Baseline	7.8 (3.3)	7.8 (3.7)	0.976
Before first brushing	4.2 (1.4)	4.0 (1)	0.783
Before last brushing	4.0 (2.2)	6.2 (3.5)	**0.017** *
*P*‐value**	**<0.001** *	**<0.001** *	
Volpe‐Manhold Index			
Baseline	9.9 (5.8)	8.9 (6.5)	0.561
Before first brushing	0 (0)	0.3 (1.3)	0.329
Before last brushing	1.5 (2.6)	6.9 (4.7)	**<0.001** *
*P*‐value**	**<0.001** *	**<0.001** *	

* *P* < 0.05 or *P* < 0.017 after alpha adjustment indicating statistical significance.

** Friedmann's non‐parametric test, two‐sided variance analysis, based on existing pre‐ and post‐teeth cases, bold numbers indicating statistical significance.

### 3.3 Vestibulum index

Regarding the investigated food debris values, no differences could be shown between treatment and control groups, nor between different time points during the study (Supplementary file 1).

### 3.4 Number of teeth and root caries index

At the end of the study compared with baseline, there was no significant difference in the number of teeth in the test group, but the number was reduced in the control group (*P* = 0.011 vs baseline). The difference between the test and control groups almost reached statistical significance in favour of brushing (*P* = 0.087). The root caries index increased in the control group at the end of the study period (*P* = 0.006), and the difference between the test and control group reached statistical significance in favour of the intervention group (*P* = 0.002; Table 3). In the intervention group, the number of lost teeth correlated with number of months living in the nursing home (*r* = 0.5, *P* = 0.035), and root caries incidence correlated with absolute number of prescribed medications (*r* = 0.5, *P* = 0.062).

**Table 3:** Number of teeth, number of lost teeth and root caries index at baseline, before first brushing and before the last brushing session

	Brushing group, mean (SD)	Controls, mean (SD)	*P*‐value
Number of teeth^a^			
Baseline	17 (8.8)	19.14 (9.25)	0.428
Before last brushing	16.7 (8.8)	18.68 (9.66)	0.518
*P*‐value**	0.157	**0.011** *	
Number of lost teeth	0.1 (0.3)	0.45 (0.91)	0.087
RCI			
Baseline	1.1 (1.2)	1.5 (1.8)	0.433
Before last brushing	1.3 (1.3)	2.6 (1.3)	**0.002** *
*P*‐value**	0.834	**0.006** *	

a Number of teeth without resident no. 19 due to special circumstances, only participants with existing pre‐ and post‐values, other cases excluded. RCI, Root Caries Index; SD, standard deviation; bold numbers indicating statistical significance.

* *P* < 0.05.

** Wilcoxon‐signed‐rank‐test.

## 4 DISCUSSION

Our hypothesis was that regular professional brushing would be efficient in maintaining or improving individual oral health status. Accordingly, we have shown the beneficial effects of regular professional brushing every 2 weeks by a dental nurse for 3 months on oral health parameters such as plaque index, gingivitis index, oral hygiene index and Volpe‐Manhold Index. We even provide some evidence of effects on long‐term parameters such as the number of teeth and the incidence of root caries. Our findings agree with those from other studies, which show that regular application of oral hygiene methods, possibly combined with fluoride application, can be successful among nursing home residents to improve oral health.^1^, ^25^, ^26^ In a study by Ekstrand et al^27^ nursing home residents were assigned to three groups: in group 1, a dental hygienist brushed teeth once a month and applied Duraphat on active root caries lesions, while in the other groups participants brushed teeth with 5000 vs 1450 ppm toothpaste twice a day. Participants who brushed with 1450 ppm showed significantly more root caries progression than the other groups. Zenthöfer et al^1^ investigated a mixture of professional cleaning and remotivation of different groups among nursing home residents with low care levels and also showed that professional teeth cleaning combined with individual instruction can improve oral hygiene, independent of whether remotivation was performed by a dentist or staff educated in dental hygiene and even when no remotivation was performed. In our study, there was also improvement in oral hygiene parameters in the control group, and there is a question whether the success in oral hygiene parameters might be due to the Hawthorne effect^28^ or at least be influenced in both groups: that is knowing they are involved in a study might lead to better brushing/supervising by staff but also by residents during the 2 weeks between brushing sessions. Also, the implementation of in‐house training—if seen as another intervention—may partly explain these improvements among the control group. However, the staff's interest in this training was very low; only one training session with a study duration of 3 months took place, and there were no changes regarding other parameters such as daily workflow and available time slots for oral hygiene or responsibilities. Thus, we doubt that these effects might have had an impact on the results. Our findings suggest a small difference regarding root caries development, shown by the RCI, and number of teeth over the short study duration of 3 months, a finding that seems surprising and might be regarded as coincidental due to the small number of teeth lost in the whole population. On the other hand, tooth loss while living in a nursing home is a well‐known, quickly‐occurring event and our result might cautiously be considered evidence of long‐term effects of oral hygiene on parameters such as the number of teeth and root caries incidence. Regularly performed oral hygiene provision not only helps to maintain good oral hygiene but ensures regular supervision of oral health. This additional effect might also lead to reduced tooth loss by early detection of teeth with treatment needs. Certainly, one might ask if it is necessary to have additional staff to brush teeth. Other studies have reported that in‐house‐solutions might include greater time provision and better training for care staff.^29^ In daily clinical practice, if daily brushing is not a practical approach because of financial and organizational restraints in a nursing home, methods should be adapted to the best possible approach in the individual setting. De Visschere et al^8^ have suggested that a priori assessment of such possibly influencing factors individually investigated for a nursing home and therefore being able to optimize the setting might lead to better success in implementing such strategies.

Participation in this study led to decreasing gingival inflammation (GI) in both groups. GI improvement was correlated with lower plaque (OHI) and staining (VMI) indices. None of these indices (GI, OHI, VMI) need additional tools/dental instruments to assess and enable easy collection of sustainable data. Regarding former studies, we propose to use these non‐invasive indices to assess data of the oral hygiene status in elderly, hospitalized patients. Gaining more detailed data (QHI or PI) did not result in additional findings. This vulnerable group of patients must not undergo unnecessary examinations, which may cause anxiety or resistance due to more invasive procedures such as staining or repetitive probing. The contradictions in our primary findings might be due to the type of plaque amount documentation in QHI (minimal amounts of plaque are stained) vs easily and visually perceptible amounts of biofilm in the OHI. The QHI was developed to document plaque control abilities in populations with good oral hygiene capabilities, contrary to our population. We therefore conclude that with high amounts of oral biofilm in this special patient population, the QHI might be an unnecessary parameter for future studies.

In our study, the xerostomic burden was very low, especially compared to other older populations.^30^, ^31^ Knowing that xerostomia is a common symptom among the elderly, with the prevalence ranging from 17% to 40% among community‐dwelling elderly people^32^ and 27%‐30% in medicated people (a statistically significant higher proportion than in non‐medicated populations),^33^, ^34^ these results do not seem reliable in light of the high number of residents diagnosed with dementia in our study. In addition, even though statistically significant differences between groups were reached, results were not of clinical relevance. However, when planning the study, it was unknown how many participants would be diagnosed with dementia, and therefore, this VAS was included. If we assume that more participants suffered from xerostomia or hyposalivation, this might also have influenced the differences in root caries that could be reported in our 3‐month study period.

There is and will be much discussion regarding the topic of delegation and task reallocation, both issues that have become increasingly relevant in medicine and dentistry.^35^, ^36^ With limited resources and a high need for oral hygiene provision, it needs to be clarified which professions should provide oral hygiene, and what are the most realistic and practical methods. This raises the question whether even untrained personal such as relatives could maintain good oral hygiene. Detailed specifications, guidelines and responsibilities would have to be developed to clarify these issues for dentists and teams. Regardless of who performs each service, there will always be the question regarding financial reimbursement and whether such a service should be combined with an oral health assessment that can control oral health and any problems that may occur. Obviously, quality assurance by a dentist in Germany is mandatory since the responsibility of the dentist cannot be delegated.

There are limitations to our study. Firstly, the 3‐month study duration is insufficient to provide results regarding long‐term efficiency. Furthermore, only one dental nurse provided all services to the nursing home residents. Regarding the external validity of this data, there might be person‐dependent differences in quality regarding the treatment success achieved. On the other hand, with the same dental nurse performing all examinations, inter‐examiner differences were eliminated. Although the dental nurse in the whole‐study procedure was not blinded, the dentist performing all clinical oral examinations was supposed to be blinded. However, due to possible conversation with the residents before and after the examinations, blinding could not always be guaranteed, which is a considerable risk of bias. Also, there might be differences between the test and control groups that were not investigated in this study, such as equipment, practices, outcome quality and daily cognitive condition of residents, which may be a potential risk of bias that should be taken into account when interpreting results. Practical experience in our study showed that residents or their caregivers or legal advisors were very positive about the study and all but one person that we asked to participate gave written informed consent. At almost every session, the first question raised was regarding costs. If residents had to pay for the procedures, there might be less enthusiasm to participate in these cleaning sessions; therefore, regarding the external validity we described an optimal scenario that might not be transferrable to a real‐life situation. There is a need for further prospective longitudinal research to better evaluate oral hygiene strategies, including different approaches regarding their feasibility and effectiveness to maintain the best possible oral health in residential care setting.

## 5 CONCLUSIONS

Regular additional professional brushing every 2 weeks by a dental nurse may be recommended for nursing homes residents to maintain and improve oral hygiene parameters as possible basis to preserve the number of teeth and reduce root caries incidence. Such oral hygiene procedures may help to maintain and improve oral health of nursing home residents.

## 6 CLINICAL RELEVANCE

### 6.1 Scientific rationale for study

The oral health of nursing home residents is poor for various reasons. Many require help for oral hygiene.

### 6.2 Principal findings

Regular additional professional brushing every 2 weeks by a dental nurse can be recommended for nursing homes residents to improve short‐term oral health parameters and to help reduce root caries incidence as a basis to preserve the number of teeth.

### 6.3 Practical implications

Such oral hygiene procedures will maintain and improve the oral health of nursing home residents.
